# Homocysteine is related to enlarged perivascular spaces in the brainstem in patients with isolated pontine infarction

**DOI:** 10.1186/s12883-022-02744-9

**Published:** 2022-08-11

**Authors:** Yunting Fu, Wenwei Yun, Zhixiang Zhang, Yi Ma, Lulu Xiao, Min Zhang, Wusheng Zhu

**Affiliations:** 1 Department of Neurology, The Affiliated Changzhou No.2 People’s Hospital of Nanjing Medical University, No.29, Xinglong Lane, Changzhou, Jiangsu Province, 213004 China; 2 Department of Radiology, The Affiliated Changzhou No.2 People’s Hospital of Nanjing Medical University, Changzhou, Jiangsu Province China; 3 Department of Neurology, Jinling Hospital, Medical School of Nanjing University, Nanjing, Jiangsu Province China; 4 Department of Neurology, Jinling Clinical Medical College of Nanjing Medical University, No.305, East Zhongshan Road, Nanjing, 210002 Jiangsu Province China

**Keywords:** Homocysteine, Cerebral small vessel disease, Enlarged perivascular spaces, Isolated pontine infarction

## Abstract

**Background:**

Homocysteine is correlated with several imaging features of cerebral small vessel disease including white matter hyperintensities, lacunes, and enlarged perivascular spaces (EPVS) in the basal ganglia. However, little is known about EPVS in the brainstem. This study aimed to investigate the correlation between serum total homocysteine (tHcy) and EPVS in the brainstem in patients with acute isolated pontine infarction.

**Methods:**

Consecutive patients with isolated pontine infarction were retrospectively enrolled. Clinical characteristics and laboratory tests including tHcy were recorded. Imaging markers of cerebral small vessel disease including EPVS in the basal ganglia (BG-EPVS), EPVS in the centrum semiovale, and EPVS in the midbrain or pons (brainstem-EPVS) were assessed using conventional magnetic resonance imaging. The relation between tHcy and EPVS of different parts in the brain was analyzed using univariate and multivariate regression model.

**Results:**

A total of 227 patients were included (mean age 67.10 ± 9.38 years, male sex 58.6%). The frequencies of brainstem-EPVS and moderate to severe BG-EPVS accounted for 40.1% (91/227) and 40.5% (92/227) respectively. After controlling for confounding factors, multivariate logistic regression analyses showed that tHcy was an independent risk factor for both moderate to severe BG-EPVS (*P* = 0.003, *P* for trend < 0.001) and the presence of brainstem-EPVS (*P* < 0.001, *P* for trend < 0.001) in a dose-dependent manner. Furthermore, multivariate linear regression model indicated that the presence of brainstem-EPVS (β = 0.264, 95% confidence interval = 0.143-0.402, *P* < 0.001) and the severity of BG-EPVS (β = 0.162, 95% confidence interval = 0.024-0.197, *P* = 0.013) were positively associated with serum tHcy.

**Conclusions:**

Serum tHcy is correlated with brainstem-EPVS and BG-EPVS dose-dependently. This study may support a contributing role for homocysteine in the pathophysiology of EPVS in the brainstem and the basal ganglia.

## Background

Homocysteine is a sulfur-containing intermediate amino acid resulting from the process of methionine metabolism [1]. Increased serum total homocysteine (tHcy) concentrations, contributing to endothelial dysfunction, have long been recognized as a risk factor for cardiovascular disease, ischemic stroke, and cognitive impairment [1–4]. While previous studies provided sufficient evidence on the causal relation between tHcy and atherosclerosis in experimental animals and ischemic stroke patients [4–6], relatively less attention has been paid to the link between tHcy and cerebral small vessel disease [7], the pathogenesis of which also involves impaired endothelial function [8].

Cerebral small vessel disease (cSVD), affecting the perforating arterioles, venules, and capillaries of the brain [9], is a complex syndrome defined by heterogeneous clinical, neuroimaging and pathological features. Although the disease is very common among the ageing population and causes extensive physical and psychological damage [8], the exact pathogenesis has not been fully elucidated [10]. Imaging features of cSVD seen on magnetic resonance imaging (MRI) mainly include white matter hyperintensities (WMH), recent small subcortical infarcts, lacunes of presumed vascular origin, cerebral microbleeds, enlarged perivascular spaces (EPVS), and brain atrophy [11].

Previous researches have focused on the correlation between serum tHcy and some components of cSVD (WMH, lacunes, cerebral microbleeds and EPVS in the basal ganglia in particular) [4, 12–16], indicating a potential role of homocysteine in the cSVD-related brain lesions. A newly published population-based Mendelian randomization study demonstrates that tHcy level is related to brain volume loss and lacunes [17]. A recent meta-analysis also supports the association of tHcy with cSVD subtypes, including WMH, silent brain infarction, and lacunar infarction [18]. However, little attention has been given to EPVS in the midbrain and pons. Whether homocysteine contributes to the pathogenesis of EPVS in the brainstem remains unknown and a relatively homogeneous population is needed to conduct further investigation.

Isolated pontine infarction (IPI) is a very common type of ischemic stroke in the brainstem [19] and EPVS in the midbrain or pons are often found in IPI patients in clinical practice, sometimes easily confused with lacunes. However, the pathogenesis and clinical implication of EPVS in the brainstem remains unclear. Therefore, this study aimed to investigate the association between serum tHcy level and EPVS in the brainstem in IPI patients, in the attempt to shed some light on the underlying pathological mechanism of EPVS in the brainstem.

## Methods

### Patients and population

Consecutive patients with acute isolated pontine infarction between January 2017 and December 2020 were retrospectively reviewed, and enrolled from a prospectively maintained stroke registry based on the neurology department of Changzhou No.2 People's Hospital (*n* = 278). Patients were included in the study if they met the following criteria: (a) age ≥ 18 years, (b) hospital admission within seven days after symptom onset, and (c) isolated pontine ischemic lesion confirmed by diffusion-weighted imaging (DWI) without extra-pontine area affected [20]. We excluded patients who did not undergo magnetic resonance angiography (MRA) or computed tomographic angiography (CTA) (*n* = 38). Patients with brain tumors (*n* = 2) and histories of brain trauma (*n* = 2) were excluded. Besides, according to Trial of Org 10172 in Acute Stroke Treatment (TOAST) criteria [21], infarctions probably originated from cardioembolism and other determined or undetermined cause were also excluded (*n* = 9). Finally, a total of 227 participants were included into the study. Approval of the study protocol was granted from the Research Ethics Committee of our hospital (ethical approval number: 2017KY015-01). Informed consent was waived because patient data were de-identified in this observational and retrospective study.

### Clinical assessment and measurement of tHcy concentration

Demographics were recorded and clinical data were extensively evaluated, including age, sex, Body Mass Index, history of ischemic stroke or transient ischemic attack (TIA) or coronary heart disease, and risk factors for ischemic stroke (hypertension, diabetes mellitus, hyperlipidemia, current smoking, alcohol use). Systolic and diastolic blood pressure on admission was recorded. Neurological deficits were assessed by National Institute of Health Stroke Scale (NIHSS) [22] on admission and at discharge.

To measure serum tHcy levels, 4-milliliter whole venous blood samples were collected in the heparin anticoagulant tubes (HengYuan Biological Technology Co., Ltd, Shanghai, China). Serum was separated by centrifugation at 4000r/min for 5 min (Eppendorf AG, Hamburg, Germany). The serum tHcy levels were determined using the ADVIA2400 automated biochemical analyzer (Siemens AG, Munich, Germany) with a standard protocol. Other laboratory tests included fasting plasma glucose (FPG), glycosylated hemoglobin (HbA1c), serum lipids (triglycerides, total cholesterol, low-density lipoprotein cholesterol (LDL-C), high-density lipoprotein cholesterol (HDL-C)), hemoglobin, fibrinogen, uric acid, creatinine, and estimated creatinine clearance (CCrE) by the Cockcroft-Gault formula [23].

### IPI classification

**Fig. 1:**
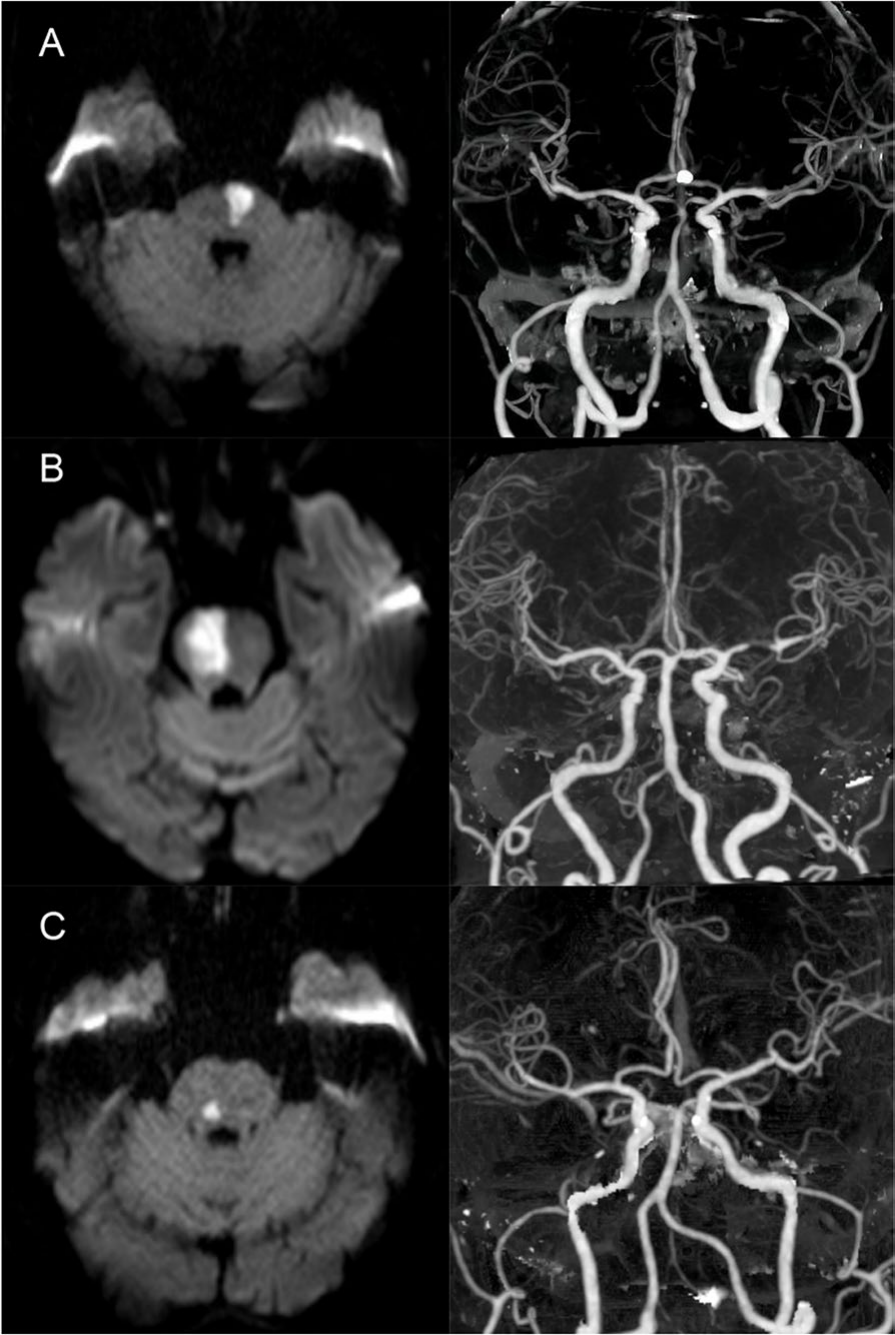
Examples of Isolated pontine infarctions. **(A)** PPI. DWI showed an infarction extending to the surface of the pons with CTA showing ≥50% stenosis in the basilar artery. **(B)** PPI. DWI showed an infarction reaching the pontine surface with irregularities of the basilar artery wall seen on CTA. **(C)** SDPI. DWI showed a lacunar infarction not reaching the basal surface of the pons without vertebrobasilar artery stenosis seen on CTA

IPI patients were categorized into two groups: paramedian pontine infarction (PPI) and small deep pontine infarction (SDPI), i.e., lacunar pontine infarction, based on lesion location and shape [19]. PPI was considered if the infarction area extended to the surface of the pontine base. SDPI was presumed if a pontine infarct did not reach the basal surface of the pons on MRI. Concerning the underlying etiology, basilar artery atherosclerosis may be a primary cause of PPI, and small vessel disease may play a major role in SDPI [24]. Examples of IPI are given in Fig. 1.

### Imaging acquisition and EPVS evaluation

All the participants underwent a 1.5-tesla magnetic resonance scanning (Achieva, Philips Medical Systems, the Netherlands) using a routine clinical brain MRI protocol. Imaging sequences and parameters were as follows: T1-weighted images (T1WI, repetition time [TR] / echo time [TE] = 263.3/6.4 milliseconds [ms], slice thickness = 5.5mm); T2-weighted images (T2WI, TR/TE = 3,592.5/100 ms, slice thickness = 5.5mm); T2 fluid-attenuated inversion recovery images (T2FLAIR, TR/TE = 4,506.7/120 ms, slice thickness = 5.5mm); diffusion weighted imaging (DWI, TR/TE = 3,002.2/75.1 ms, slice thickness=6.6 mm). Three-dimensional contrast-enhanced MRA was performed for 50 patients (TR/TE = 5.6/1.9 ms, slice thickness = 1.4 mm). CTA was performed for the other 177 patients with dual-source CT system (SOMATOM Definition Flash, Siemens, Forchheim, Germany).

**Fig. 2:**
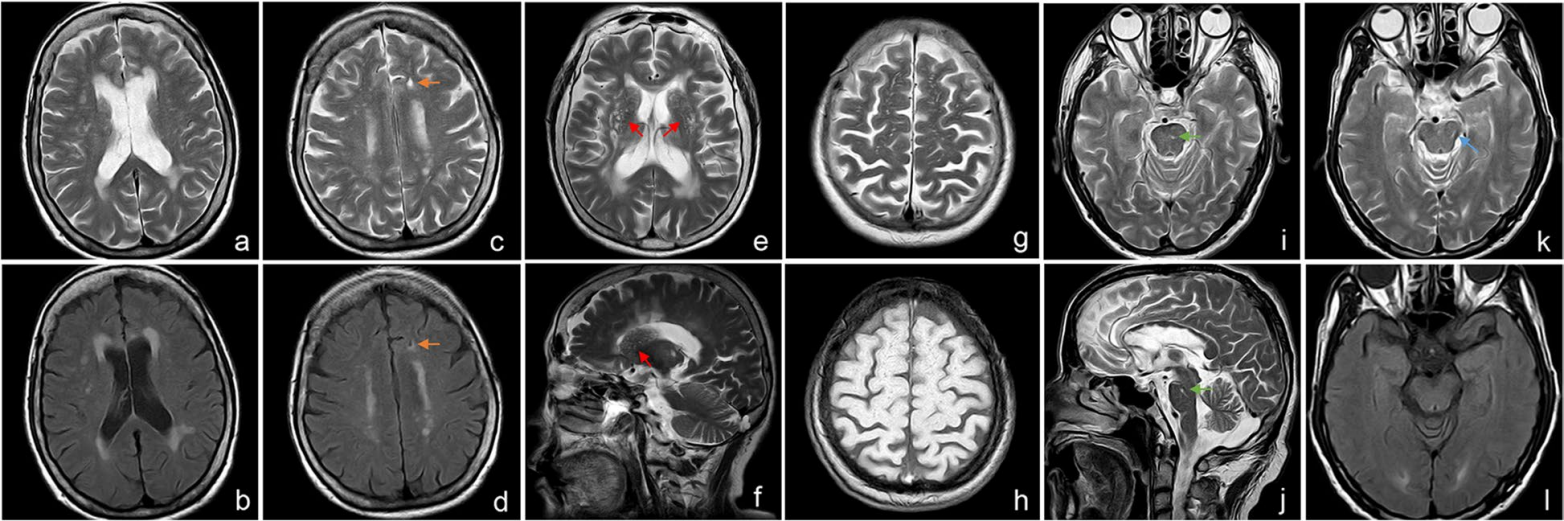
Imaging features of cSVD in IPI patients. **(a-d)** T2 and FLAIR axial images were from a female patient aged 74 years. **(a-b)** Moderate WMH with a Fazekas score of 3. **(c-d)** One lacune of presumed vascular origin located in the left subcortical area (orange arrows). **(e-f)** T2 axial and sagittal images were from a female patient aged 73 years, showing moderate to severe BG-EPVS with a visual score of 3 (red arrows). **(g-h)** T2 and T1 axial images were from a 69-year-old female patient, showing moderate to severe CSO-EPVS with a visual score of 3. **(i-j)** T2 axial and sagittal images were from a 64-year-old male patient, showing brainstem-EPVS in the left medial pons (green arrows). **(k-l)** T2 and FLAIR axial images were from a male patient aged 61 years, showing brainstem-EPVS in the left lateral midbrain (blue arrow)

Imaging markers of cSVD on MRI were under careful assessment by two experienced neurologists (MZ, ZXZ) who were blinded to patients’ clinical data. Any disagreement between the two observers was settled by discussion with another senior neurologist (WWY). We defined enlarged perivascular spaces (EPVS) as < 3mm round or linear lesions surrounding perforating vessels with a CSF-like signal intensity (T1/FLAIR-hypointense and T2-hyperintense). Typical brain regions of EPVS include the basal ganglia (BG), the centrum semiovale (CSO), the midbrain, and the pons. BG-EPVS and CSO-EPVS were rated respectively with a validated visual scale from 0 to 4 based on the hemispherical slice showing the greatest numbers of EPVS (0 = none, 1 = 1-10, 2 = 11-20, 3 = 21-40, and 4 = > 40) [25]. We considered the severity of EPVS as moderate to severe when the score ranged from 2-4 in BG or CSO. Brainstem-EPVS, combining EPVS in the midbrain and the pons, were scored 0 (none visible) or 1 (visible) [25]. Inter and intra rater reliability for the evaluation of EPVS, using 50 randomly selected scans, was estimated by Kappa test, which showed good agreement (Brainstem-EPVS: κ = 0.72 & 0.76; BG-EPVS: κ = 0.82 & 0.88; CSO-EPVS: κ = 0. 79 & 0.85). The presence of lacunes was recorded and carefully distinguished from EPVS. Lacunes of presumed vascular origin [11], having a round or ovoid shape, filled with CSF-like fluid, were located in the territory of perforating arterioles. Lacunes generally measure 3-15mm in diameter on T1WI or T2WI sequences, with perilesional hyperintense rims on FLAIR sequences. WMH present as bilateral hyperintensities in periventricular and deep white matter on T2WI or FLAIR sequences and WMH grade was assessed with the Fazekas scale [26]. Moderate to severe WMH was defined when the total score ranged from 3 to 6. (Fig. 2)

### Statistical analysis

Quantitative data were tested for normal distribution with the Shapiro-Wilk test. Normally distributed continuous variables were expressed as mean ± standard deviation, and those with skewed distribution were presented as median (interquartile range). Categorical variables were summarized as numbers (percentages). Patients were dichotomized into the lower and higher tHcy groups based on the median value of serum tHcy concentrations. To compare the characteristics between the two groups, Student’s *t*-test or Mann-Whitney *U* test was used for continuous data and χ^2^ tests or Fisher’s exact tests were used for categorical ones where appropriate. We applied binary logistic regression model to identify risk factors for moderate to severe BG-EPVS, CSO-EPVS and the presence of brainstem-EPVS. Simple and multiple linear regression analyses were conducted to assess the association between serum tHcy and underlying factors, in which tHcy levels were ln-transformed due to non-normal distribution. Parameters with a *P* value < 0.05 in univariate analyses were finally included in the multivariate logistic and linear regression analyses. According to the variance inflation factor (VIF), the multicollinearity test indicated that no evidence of collinearity existed among the independent variables (the mean VIF = 1.988, the maximum VIF = 3.779, and the minimum VIF = 1.210). Additional multivariate logistic regression analysis was performed to investigate detailed information regarding the association between serum tHcy quartiles and BG-EPVS severity or the presence of brainstem-EPVS. The *P* value for trend across the quartiles of tHcy was also determined in the process. All statistical analyses were carried out using SPSS, version 22.0 (IBM Corp., Armonk, NY). Two- sided *P* values < 0.05 were considered statistically significant.

## Results

### Demographic and clinical characteristics of participants

A total of 227 IPI patients were enrolled in this study (mean age 67.10 ± 9.38 years, male sex 58.6%). The frequency of brainstem-EPVS was 40.1% (91/227), including the pons 18.5% (42/227), midbrain 15.4% (35/227), and both the pons and midbrain 6.2% (14/227). The frequency of moderate to severe BG-EPVS was 40.5% (92/227). The rate of moderate to severe WMH was also 40.1% (91/227). The presence of lacunes accounted for a higher rate, which was 55.9% (127/227). The median level of serum tHcy was 12.46 (9.49, 17.40) μmol/L.

The baseline characteristics of the lower and higher tHcy groups classified according to the median level are displayed in Table 1. Patients with higher serum tHcy had a higher rate of current smoking, moderate to severe BG-EPVS, brainstem-EPVS, and higher levels of uric acid than patients with lower tHcy level (all *P* <0.05). No significant difference in other risk factors for stroke, NIHSS score, IPI classification, moderate to severe WMH or CSO-EPVS, and the presence of lacunes was found between the two groups.

**Table 1:** Demographics and baseline characteristics between lower and higher tHcy groups

Items	lower tHcy group (<12.46μmol/L)	higher tHcy group (≥12.46μmol/L)	***P*** value
Number	113	114	
Sex, male	61 (54.0%)	72 (63.2%)	0.161
Age, years	66.90 ± 8.69	67.30 ± 10.04	0.751
Body Mass Index, kg/m^2^	23.42 (22.22, 24.87)	23.52 (22.37, 25.95)	0.189
Hypertension	107 (94.7%)	100 (87.7%)	0.064
SBP, mmHg	152.04 ± 20.45	152.32 ± 20.23	0.920
DBP, mmHg	85.08 ± 10.31	83.74 ± 10.97	0.343
Diabetes	75 (66.4%)	69 (60.5%)	0.361
FPG, mmol/L	6.17 (5.29, 8.66)	5.98 (5.31, 8.81)	0.724
HbA1c, %	6.90 (6.00, 8.85)	6.55 (5.90, 8.90)	0.564
Ischemic stroke or			
TIA history	24 (21.2%)	27 (23.7%)	0.659
Coronary heart disease	5 (4.4%)	10 (8.8%)	0.187
Current smoking	33 (29.2%)	50 (43.9%)	0.022*
Alcohol use	18 (15.9%)	19 (16.7%)	0.880
Hyperlipidemia	40 (35.7%)	36 (31.6%)	0.511
Total cholesterol, mmol/L	4.39 ± 1.15	4.40 ± 0.89	0.972
Triglyceride, mmol/L	1.48 (1.07, 2.28)	1.57 (1.13, 2.00)	0.995
HDL-C, mmol/L	1.04 (0.88, 1.24)	1.01 (0.85, 1.19)	0.251
LDL-C, mmol/L	2.42 ± 0.85	2.50 ± 0.68	0.453
Hemoglobin, g/L	137.00 (127.50, 149.00)	139.50 (129.75, 147.25)	0.508
Fibrinogen, g/L	2.96 (2.49, 3.41)	2.85 (2.38, 3.44)	0.472
CCrE, mL/(min*1.73m^2^)	88.87 (71.30, 101.51)	80.48 (63.06, 101.52)	0.116
Uric acid, μmol/L	284.00 (234.45, 337.25)	308.10 (262.78, 374.00)	0.013*
NIHSS at baseline	3 (2, 5)	3 (2, 5)	0.790
NIHSS at discharge	3 (2, 5)	3 (2, 5)	0.823
IPI classification			0.129
PPI	76 (67.3%)	87 (76.3%)	
SDPI	37 (32.7%)	27 (23.7%)	
Moderate to severe WMH	48 (42.5%)	43 (37.7%)	0.464
Lacunes	61 (54.0%)	66 (57.9%)	0.553
CSO-EPVS grade			0.261
0	5 (4.4%)	1 (0.9%)	
1	52 (46.0%)	46 (40.4%)	
2	36 (31.9%)	38 (33.3%)	
3	13 (11.5%)	22 (19.3%)	
4	7 (6.2%)	7 (6.1%)	
Moderate to severe			
CSO-EPVS	56 (49.6%)	67 (58.7%)	0.164
BG-EPVS grade			
0	1 (0.9%)	0 (0.0%)	<0.001*
1	82 (72.6%)	52 (45.6%)	
2	25 (22.1%)	37 (32.5%)	
3	4 (3.5%)	24 (21.0%)	
4	1 (0.9%)	1 (0.9%)	
Moderate to severe			
BG-EPVS	30 (26.5%)	62 (54.4%)	<0.001*
Brainstem-EPVS	24 (21.2%)	67 (58.8%)	<0.001*

*Abbreviations*: *tHcy* total homocysteine, *SBP* systolic blood pressure, *DBP* diastolic blood pressure, *FPG* fasting plasma glucose; *HbA1c* glycosylated hemoglobin, *TIA* transient ischemic attack, *HDL-C* high-density lipoprotein cholesterol, *LDL-C* low-density lipoprotein cholesterol, *CCrE* estimated creatinine clearance, *NIHSS* National Institute of Health Stroke Scale, *IPI* isolated pontine infarction, *PPI* paramedian pontine infarction, *SDPI* small deep pontine infarction, *WMH* white matter hyperintensities, *EPVS* enlarged perivascular spaces, *CSO* the centrum semiovale, *BG* the basal ganglia

Data presented as n (%), mean ± standard deviation, or median (interquartile range)

* *P* < 0.05

### EPVS of different locations in the brain and possible risk factors

In univariate and multivariate logistic regression analyses (Tables 2-3), tHcy (adjusted odds ratio [aOR] = 1.048, 95% confidence interval [CI] = 1.016-1.082, *P* = 0.003) and WMH grade (aOR = 1.380, 95% CI = 1.103-1.727, *P* = 0.005) were independent risk factors for moderate to severe BG-EPVS. However, no underlying factors showed correlation with CSO-EPVS. Ischemic stroke or TIA history (aOR = 2.874, 95% CI = 1.402-5.891, *P* = 0.004) and tHcy (aOR = 1.099, 95% CI = 1.051-1.149, *P* < 0.001) remained influencing factors for brainstem-EPVS. Moreover, higher quartile of tHcy was significantly associated with moderate to severe BG-EPVS and the presence of brainstem-EPVS dose-dependently after adjusting for confounding factors (both *P* value for trend < 0.001). As shown in Table 4, compared to the lowest quartile of tHcy, the top tHcy quartile was significantly associated with increased frequency of moderate to severe BG-EPVS (aOR = 5.437, 95% CI = 2.188-13.511, *P* < 0.001) and the presence of brainstem-EPVS (aOR = 8.949, 95% CI = 3.544-22.592, *P* < 0.001).

**Table 2:** Univariable logistic regression analyses between possible factors and EPVS of three locations in the brain

Factors	moderate to severe BG-EPVS		moderate to severe CSO-EPVS		brainstem-EPVS	
	OR (95% CI)	***P*** value	OR (95% CI)	***P*** value	OR (95% CI)	***P*** value
Sex, male	0.921 (0.537-1.578)	0.763	1.005 (0.591-1.707)	0.986	0.700 (0.406-1.206)	0.199
Age	1.057 (1.024-1.091)	0.001*	0.988 (0.960-1.016)	0.390	1.020 (0.991-1.050)	0.172
Body Mass Index	0.959 (0.869-1.057)	0.397	1.034 (0.940-1.138)	0.489	0.961 (0.871-1.060)	0.427
Hypertension	1.658 (0.613-4.488)	0.319	1.202 (0.480-3.011)	0.694	0.515 (0.205-1.299)	0.160
SBP	1.010 (0.997-1.024)	0.124	0.998 (0.985-1.011)	0.766	1.001 (0.988-1.014)	0.875
DBP	0.983 (0.958-1.008)	0.182	0.988 (0.964-1.013)	0.349	0.988 (0.963-1.013)	0.347
Diabetes	1.138 (0.655-1.978)	0.646	1.077 (0.627-1.852)	0.788	0.807 (0.466-1.397)	0.443
FPG	0.927 (0.842-1.022)	0.127	0.995 (0.909-1.089)	0.916	1.022 (0.932-1.120)	0.646
HbA1c	0.935 (0.828-1.057)	0.283	1.013 (0.901-1.140)	0.829	1.010 (0.896-1.138)	0.872
Hyperlipidemia	0.662 (0.373-1.174)	0.158	0.765 (0.440-1.330)	0.342	1.154 (0.658-2.023)	0.618
Total cholesterol	0.745 (0.567-0.980)	0.035*	0.902 (0.698-1.165)	0.427	1.134 (0.875-1.471)	0.341
Triglyceride	0.708 (0.529-0.946)	0.020*	0.896 (0.721-1.115)	0.326	0.845 (0.661-1.081)	0.179
HDL-C	1.634 (0.592-4.510)	0.343	1.347 (0.491-3.696)	0.562	2.591 (0.923-7.276)	0.071
LDL-C	0.697 (0.486-1.000)	0.050	0.997 (0.709-1.404)	0.988	1.239 (0.875-1.756)	0.227
Ischemic stroke or TIA history	2.130 (1.133-4.005)	0.019*	1.573 (0.829-2.984)	0.165	3.340 (1.747-6.386)	<0.001*
Coronary heart disease	1.307 (0.457-3.739)	0.617	1.289 (0.443-3.751)	0.641	0.996 (0.342-2.901)	0.994
Current smoking	0.749 (0.429-1.306)	0.308	1.168 (0.678-2.012)	0.575	1.341 (0.774-2.321)	0.295
Alcohol use	1.001 (0.488-2.050)	0.999	1.292 (0.632-2.643)	0.482	1.972 (0.969-4.012)	0.061
Fibrinogen	1.187 (0.819-1.719)	0.365	0.989 (0.686-1.425)	0.952	1.407 (0.967-2.048)	0.075
CCrE	0.982 (0.970-0.995)	0.006*	1.009 (0.997-1.022)	0.141	0.987 (0.974-0.999)	0.036*
Uric acid	1.000 (0.997-1.003)	0.881	1.001 (0.998-1.004)	0.634	1.004 (1.001-1.007)	0.019*
tHcy	1.047 (1.015-1.079)	0.003*	0.996 (0.971-1.022)	0.773	1.104 (1.057-1.152)	<0.001*
WMH grade	1.48 (1.210-1.811)	<0.001*	1.063 (0.884-1.278)	0.519	1.223 (1.012-1.477)	0.037*
Lacunes	1.632 (0.950-2.804)	0.076	1.352 (0.798-2.289)	0.262	1.844 (1.068-3.183)	0.028*
IPI classification	1.089 (0.603-1.966)	0.778	1.379 (0.772-2.463)	0.277	0.810 (0.451-1.455)	0.481

*Abbreviations*: *tHcy* total homocysteine, *SBP* systolic blood pressure, *DBP* diastolic blood pressure, *FPG* fasting plasma glucose, *HbA1c* glycosylated hemoglobin, *TIA* transient ischemic attack, *HDL-C* high-density lipoprotein cholesterol, *LDL-C* low-density lipoprotein cholesterol, *CCrE* estimated creatinine clearance, *IPI* isolated pontine infarction, *PPI* paramedian pontine infarction, *SDPI* small deep pontine infarction, *WMH* white matter hyperintensities, *EPVS* enlarged perivascular spaces, *CSO* the centrum semiovale, *BG* the basal ganglia, *OR* odds ratio, *CI* confidence interval

WMH grade was assessed on T2FLAIR sequences with the Fazekas scale ranging from 0-6

* *P* < 0.05

**Table 3:** Multivariable logistic regression analyses between possible factors and BG-/brainstem-EPVS

Factors	moderate to severe BG-EPVS ^**a**^		brainstem-EPVS ^**b**^	
	adjusted OR (95% CI)	***P*** value	adjusted OR (95% CI)	***P*** value
Age	1.033 (0.988-1.079)	0.153	—	—
Total cholesterol	0.782 (0.566-1.080)	0.135	—	—
Triglyceride	0.818 (0.601-1.112)	0.200	—	—
Ischemic stroke or TIA history	1.814 (0.897-3.667)	0.097	2.874 (1.402-5.891)	0.004*
CCrE	0.996 (0.979-1.013)	0.629	0.995 (0.981-1.009)	0.488
Uric acid	—	—	1.003 (0.999-1.006)	0.150
tHcy	1.048 (1.016-1.082)	0.003*	1.099 (1.051-1.149)	<0.001*
WMH grade	1.380 (1.103-1.727)	0.005*	1.213 (0.973-1.513)	0.087
Lacunes	—	—	1.306 (0.691-2.471)	0.411

*Abbreviations*: *tHcy* total homocysteine, *TIA* transient ischemic attack, *CCrE* estimated creatinine clearance, *WMH* white matter hyperintensities, *EPVS* enlarged perivascular spaces, *BG* the basal ganglia, *OR* odds ratio, *CI* confidence interval

^a^ Adjusted with P < 0.05 in the univariable analysis (age, total cholesterol, triglyceride, ischemic stroke or TIA history, CCrE, and WMH grade)

^b^ Adjusted with P < 0.05 in the univariable analysis (ischemic stroke or TIA history, CCrE, uric acid, WMH grade, and lacunes of presumed vascular origin)

* *P* < 0.05

**Table 4:** The association between serum tHcy and BG-/brainstem-EPVS

serum tHcy (μmol/L)	BG-EPVS		adjusted OR (95% CI) ^**a**^	***P*** value	brainstem-EPVS		adjusted OR (95% CI) ^**b**^	***P*** value
	mild	moderate to severe			none	visible		
Quartile 1 (≤9.49)	43 (75.4%)	14 (24.6%)	1.000 (reference)	—	43 (75.4%)	14 (24.6%)	1.000 (reference)	—
Quartile 2 (9.50-12.46)	41 (71.9%)	16 (28.1%)	1.123 (0.453-2.785)	0.803	47 (82.5%)	10 (17.5%)	0.520 (0.196-1.380)	0.189
Quartile 3 (12.47-17.40)	29 (50.9%)	28 (49.1%)	3.485 (1.447-8.395)	0.005*	32 (56.1%)	25 (43.9%)	2.369 (1.005-5.583)	0.049*
Quartile 4 (≥17.41)	22 (39.3%)	34 (60.7%)	5.437 (2.188-13.511)	<0.001*	14 (25.0%)	42 (75.0%)	8.949 (3.544-22.592)	<0.001*
*P* for trend				<0.001*				<0.001*

*Abbreviations*: *tHcy* total homocysteine, *EPVS* enlarged perivascular spaces, *BG* the basal ganglia, *OR* odds ratio, *CI* confidence interval

^a^ Adjusted for age, total cholesterol, triglyceride, ischemic stroke or TIA history, CCrE, and WMH grade

^b^ Adjusted for ischemic stroke or TIA history, CCrE, uric acid, WMH grade, and lacunes of presumed vascular origin

Data presented as n (%)

* *P* < 0.05

### Serum tHcy and EPVS in the brainstem

To further elucidate the relationship between tHcy and EPVS of different parts in the brain, we transformed tHcy into a natural logarithmic scale and performed univariable and multivariable linear regression analyses. In simple linear regression model, the presence of brainstem-EPVS was positively associated with serum tHcy (standardized β = 0.367, 95% CI = 0.253-0.506, *P* < 0.001). In multiple linear regression model, Table 5 demonstrated that the presence of brainstem-EPVS (standardized β = 0.264, 95% CI = 0.143-0.402, *P* < 0.001), the severity of BG-EPVS (standardized β = 0.162, 95% CI = 0.024-0.197, *P* = 0.013), and current smoking (standardized β = 0.170, 95% CI = 0.025-0.333, *P* = 0.023) were positively correlated with serum tHcy, adjusting for sex, age, and all the confounding factors identified in the univariate linear regression model.

**Table 5:** Multivariable linear regression full model for tHcy ^c, d^

variable	B	standardized β	95%CI	***P*** value
Sex, male	-0.138	-0.134	-0.295-0.019	0.085
Age	-0.001	-0.015	-0.009-0.008	0.855
SBP	0.002	0.074	-0.002-0.006	0.329
DBP	-0.001	-0.026	-0.008-0.006	0.733
FPG	-0.038	-0.217	-0.077-0.000332	0.052
HbA1c	0.014	0.061	-0.036--0.064	0.585
Current smoking	0.179	0.170	0.025-0.333	0.023*
CCrE	-0.002	-0.103	-0.006-0.001	0.183
Uric acid	0.00042	0.076	-0.000291-0.001	0.245
BG-EPVS grade	0.110	0.162	0.024-0.197	0.013*
brainstem-EPVS	0.273	0.264	0.143-0.402	<0.001*

*Abbreviations*: *tHcy* total homocysteine, *SBP* systolic blood pressure, *DBP* diastolic blood pressure, *FPG* fasting plasma glucose, *HbA1c* glycosylated hemoglobin, *CCrE* estimated creatinine clearance, *EPVS* enlarged perivascular spaces, *BG* the basal ganglia, *CI* confidence interval

^c^ tHcy levels were transformed to a natural logarithmic scale

^d^ Age, sex, and other factors with *P* < 0.05 in simple linear regression analyses were included in the multivariate model

* *P* < 0.05

## Discussion

In this study, we found that serum tHcy concentration was related to EPVS in the brainstem in patients with acute isolated pontine infarction. Multivariable logistic analyses showed that tHcy was an independent risk factor for both the presence of brainstem-EPVS and moderate to severe BG-EPVS in a dose-dependent manner. Moreover, based on the results of simple and multiple linear regression analyses, it is estimated that about 28.1% of the association between brainstem-EPVS and tHcy levels was explained by other influencing factors mainly including the coexistence of BG-EPVS. ((0.367-0.264) / 0.367 ≈ 28.1%)

In our study, higher percentage of participants with moderate to severe BG-EPVS or the presence of brainstem-EPVS was identified in the higher tHcy group (≥12.46μmol/L). Although the underlying mechanisms of EPVS remain unclear, our study may provide evidence that tHcy contributes to the development of brainstem-EPVS and increased burden of BG-EPVS visible on conventional MRI scans. Abnormal elevation of serum tHcy concentration, generally over 15μmol/L, is defined as hyperhomocysteinemia [27], which is caused or influenced by various factors including genetic variation, nutritional deficiency, and certain kinds of metabolic disorders or drugs [28]. Accumulating experimental and clinical evidence demonstrates that hyperhomocysteinemia induces endothelial dysfunction, triggers oxidative stress, and precipitates neuroinflammation [7, 29–31]. Thus, through the complicated homocysteine-mediated damaging process, one of the major consequences is blood- brain barrier (BBB) impairment [32]. More specifically, a possible explanation of the positive link between tHcy and the development of EPVS in the brain is as follows. Elevation of serum tHcy not only inhibits the normal functioning of BBB, but also destroys its structural composition [33, 34], resulting in excessive fluid in the perivascular spaces and interfering with the glymphatic system while clearing interstitial fluid [35, 36].

Recent clinical studies have found that tHcy level is related to BG-EPVS [15, 16], but not to CSO-EPVS [16], suggesting that EPVS at different sites might have different pathogenesis. Consistent with previous researches, there was no association of the severity of CSO-EPVS with serum tHcy and other vascular risk factors in our study. Studies in rodents and humans have also confirmed that CSO-EPVS and BG-EPVS differ in anatomical structures. The basal perivascular spaces, directly communicating with the subarachnoid space, are between two layers of leptomeninges which surround perforating arterioles in the basal ganglia [37]. In contrary, cortical arterioles and all venules are invested only by one layer of pia mater, and the cortical perivascular spaces are thought to connect to subpial space [38]. Whether CSO-EPVS communicating with subarachnoid space or not is still controversial [39]. In terms of draining interstitial fluid that is formed during the fluid exchange process at the BBB [40] and that contains waste products from metabolic activities in the brain, it remains unclear whether the basal and cortical perivascular spaces differ in the precise mechanism or specific fluid drainage pathways [35].

To date, the pathogenesis of EPVS in the midbrain and the pons is not elucidated. In our study, elevated serum tHcy level was a shared influencing factor for the presence of brainstem-EPVS and the severity of BG-EPVS. Thus, we propose a speculation that the formation of EPVS in the brainstem may share some common mechanism with that of EPVS in the basal ganglia and that higher levels of serum tHcy might be involved in the pathogenesis of both brainstem-EPVS and BG-EPVS. From an anatomical point of view, the blood supplies for both the brainstem and the basal ganglia arise from perforating arteries in the posterior or anterior circulation. The classical theory considers these perforators as end-vessels and the pathophysiology of small vessel disease is related to small perforating arteries and arterioles in the brain [41]. Based on the vascular pathologies of these perforators that manifest as thickening of the media [42], on the one hand, the brainstem would develop ischemic lesions. On the other hand, elevated concentration of tHcy might be involved in the process of endothelial injury and BBB leakage in the brainstem, contributing to the formation of MRI-visible EPVS. In the future, more advanced in-vivo imaging researches are needed to facilitate the detailed understanding of the anatomy of EPVS in the brainstem and the basal ganglia.

In our study, apart from tHcy, WMH were also associated with moderate to severe BG-EPVS, which is in accordance with previous studies [10]. By contrast, a history of ischemic stroke or TIA was linked to brainstem-EPVS. These findings suggest that the associations of EPVS vary with perivascular space location, which may be a reflection of the difference in the functioning of perivascular spaces [35]. While perivascular spaces play an important role in interstitial fluid drainage and waste clearance, the precise pathways are still unclear. Further researches are needed to clarify the exact drainage pathways in which brainstem-EPVS and BG-EPVS are involved. Of note, some caution is required in the interpretation of our findings because there may be confounding factors in the association between a history of ischemic stroke and brainstem-EPVS.

Although previous randomized trials have not shown benefit of vitamin B supplementation in preventing recurrence of stroke [43], it remains uncertain whether homocysteine-lowering therapy is effective in reducing the progression of cSVD or some subtypes of cSVD. A subgroup analysis of the VITAmins TO Prevent Stroke (VITATOPS) trial showed that administering B vitamins daily to recent ischemic stroke patients with severe cSVD significantly reduced the progression of WMH, but not the incidence of lacunes [44]. Evidence from other high-quality clinical interventional studies is scarce. Further prospective studies with large sample sizes are also needed to examine whether homocysteine-lowering strategy can slow the progression of EPVS in the basal ganglia and the brainstem.

There were several limitations to our study. First, it was a retrospective, cross-sectional study which could not establish a causal relationship. Second, although including only IPI patients facilitates the homogeneity of the study population for reducing potential confounding factors, it restricts the generalizability of our findings. Further prospective studies including patients with other types of cerebrovascular diseases and healthy controls are needed. Third, we did not assess cerebral microbleeds due to a lack of T2 gradient echo images in the routine MRI protocol. Fourth, other confounding factors that affect serum tHcy concentration such as serum vitamin B_12_ levels, folate levels, and mutations of methylenetetrahydrofolate reductase (MTHFR) gene were not considered in the analysis, except for smoking status. Finally, this study was unable to demonstrate significantly positive associations of tHcy level with WMH grade and the presence of lacunes in patients with acute isolated pontine infarction. The inconsistency with previous researches may be attributable to different characteristics of the study population. About 90% of the patients in our study had hypertension. Therefore, the variation of WMH burden and lacunes, which has a close relation with hypertension, was not high enough to generate significant results.

## Conclusion

This study showed that an elevated serum tHcy concentration was significantly linked to both the presence of brainstem-EPVS and the severity of BG-EPVS in IPI patients. These findings indicate that tHcy may contribute to the pathogenesis of EPVS in the brainstem and the basal ganglia.

## Data Availability

The data that support the findings of this study are available from the corresponding authors upon reasonable request.

## Abbreviations

tHcy: total Homocysteine
cSVD: Cerebral small vessel disease
MRI: Magnetic resonance imaging
WMH: White matter hyperintensities
EPVS: Enlarged perivascular spaces
IPI: Isolated pontine infarction
PPI: Paramedian pontine infarction
SDPI: Small deep pontine infarction
DWI: Diffusion-weighted imaging
MRA: Magnetic resonance angiography
CTA: Computed tomographic angiography
TIA: Transient ischemic attack
NIHSS: National Institute of Health Stroke Scale
SBP: Systolic blood pressure
DBP: Diastolic blood pressure
FPG: Fasting plasma glucose
HbA1c: Glycosylated hemoglobin
LDL-C: Low-density lipoprotein cholesterol
HDL-C: High-density lipoprotein cholesterol
CCrE: Estimated creatinine clearance
BG: the Basal ganglia
CSO: the Centrum semiovale
aOR: Adjusted odds ratio
CI: Confidence interval
BBB: Blood-brain barrier
